# Evolutionary Analysis of Prefabrication Implementation in Construction Projects under Low-Carbon Policies

**DOI:** 10.3390/ijerph191912511

**Published:** 2022-09-30

**Authors:** Qianqian Shi, Ziyu Wang, Boya Li, Marcel Hertogh, Shuyi Wang

**Affiliations:** 1 College of Economics and Management, Nanjing University of Aeronautics and Astronautics, Nanjing 211106, China; 2 Research Centre for Soft Energy Science, Nanjing University of Aeronautics and Astronautics, Nanjing 211106, China; 3 Faculty of Civil Engineering and Geosciences, Delft University of Technology, 2628 CN Delft, The Netherlands; 4 School of Civil Engineering, Southeast University, Nanjing 211189, China; 5 Department of Civil and Environmental Engineering, National University of Singapore, Singapore 119077, Singapore

**Keywords:** green construction, prefabrication, low-carbon policy, evolutionary game

## Abstract

In the context of carbon peak and carbon neutral policies, low-carbon construction has been the focus of most countries worldwide. As one of the most effective ways to achieve green construction, many countries have launched low-carbon policies to promote the development of prefabrication. However, the effectiveness and influencing factors of low-carbon policies on prefabrication need to be further verified under the dynamic game between the government and the construction enterprise. Therefore, this study considered subsidy and carbon tax policies and developed an evolutionary game model to promote the development of the prefabricated construction market. The evolutionary stable strategy of the government and construction enterprise under different scenarios was obtained. Subsequently, a numerical analysis was conducted to further investigate the impact of the key factors on the stable strategy. The results showed that an appropriate hybrid policy of subsidies and taxes could positively promote the prefabrication implementation of the construction enterprise. The government should adopt an appropriate policy intensity according to the maturity of the market. This study can provide effective guidance and practical enlightenment for the government to achieve low-carbon, green, and sustainable construction.

## 1. Introduction

### 1.1. Background

Conventional construction projects account for a very high proportion of the environmental load in global economic and social activities owing to their high consumption of natural resources, solid waste production, and carbon dioxide emissions. The 2021 Global Status Report for Buildings and Construction shows that the construction industry accounted for 36% and 37% of the global terminal energy consumption and energy-related carbon dioxide emissions in 2020, respectively. As a result, increasing the level of energy efficiency and low-carbon transformation in the building sector has attracted worldwide attention. For example, the European Union has revised its Emissions Reduction Sharing Regulation, the UK has proposed a “zero building” plan, and the Japanese government has published “Measures and Implementation Methods for Carbon Neutral Housing and Buildings by 2050”. Currently, the government’s low-carbon policy focuses on carbon neutrality [1,2], green recovery plans [3], developing emerging industries [4], and promoting the decarbonisation process [5]. In 2020, China put forward the policy goal of a carbon peak in 2030 and carbon neutrality in 2060 [6]. The country also proposed implementing tax incentives for environmental protection, energy and water conservation, new energy and clean energy vehicles and vessels, and improving “double carbon” fiscal policies. In accordance with the current urbanisation process and the situation of China’s construction industry, China’s construction industry may reach the carbon peak in 2035, five years later than the national plan [7]. Therefore, it is of great significance to choose green and low-carbon construction methods to realise a low-carbon construction industry, which is significant in mitigating climate change and saving natural resources.

In prefabrication, building components are manufactured in factories and transported to the construction site for installation. This approach improves construction efficiency and quality, and reduces labour requirements and resource consumption [8,9,10,11,12,13,14,15,16]. Teng et al. [17] found that prefabricated buildings achieved an average of 15.6% embodied carbon reduction and 3.2% operational carbon reduction compared to conventional buildings. Tumminia et al. [18] and Li et al. [19] reported that prefabrication could effectively reduce carbon emissions and the consumption of environmental value. Given the advantages of prefabrication in energy efficiency, governments have introduced numerous policies to actively promote prefabricated buildings. For example, the United States has adopted incentives such as energy efficiency funds, cash subsidies, and tax credits. Moreover, France has implemented the Universal Building System, Singapore has built an Ease of Construction Scoring System, and Malaysia has implemented the Industrial Building System (IBS) Strategy plan. As environmental and energy issues become increasingly severe, the Chinese government has introduced corresponding policies for prefabricated construction projects. In 2013, the Green Building Action Plan focused on developing prefabricated buildings, and in 2015, the assessment standards for industrial buildings were adopted to support the transformation of traditional building methods into prefabricated building methods. The proportion of prefabricated buildings in new buildings and the level of prefabricated construction have become the focus of national policies.

Scholars have conducted in-depth studies on how governments can promote green construction and prefabrication. Currently, incentive policies for prefabricated production include financial subsidies, tax incentives, floor space incentives, loan support, land supply, mandatory policies, and non-economic incentives [20]. For prefabricated practitioners, financial incentives by the government can effectively reduce the burden on families [21]. Li and Zhang [22] used externality theory and cost analysis to reveal that financial subsidies and land incentives could effectively motivate developers to achieve green construction. If the financial incentives do not offset the additional construction costs and construction enterprises do not benefit sufficiently from the prefabricated construction techniques, developers will continue to use traditional construction methods. Du et al. [23] pointed out that financial support for green construction is minimal and that most incentives are not combined with energy efficiency and emission reduction policies, lowering the development of green construction. In addition to financial support, Yu et al. [24] argued that non-financial incentives should also be considered to encourage stakeholders to adopt the prefabricated production method. Most studies on prefabrication implementation have mainly concentrated on technical applications [25,26], cost-effectiveness [27,28], and sustainability [29]. However, little attention has been paid to evaluating the influence of the mixed low carbon and subsidy policy from the government on prefabrication implementation.

### 1.2. Research Purpose and Framework

In order to fill this gap and maximise the overall comprehensive benefits of the exploration, reveal and guide the policy implementation, and promote active adoption of prefabricated production by construction enterprises, an evolutionary game model is constructed in this study to answer the following questions. How do the mixed policies of a carbon tax and subsidy dynamically affect the decision-making process of construction enterprises? How do the key factors affect the evolutionary behaviours of the government and construction enterprises? The research presented herein can reveal the dynamic game process between the government and construction enterprises, provide practical decision-making guidance for the government and construction enterprises, and promote high-quality development of green construction.

The remainder of this paper is as follows. Section 2 describes the problems and presents the basic assumptions and corresponding parameters and variables. Subsequently, an evolutionary game model is constructed, and the probable scenarios of an equilibrium state are analysed. Next, a numerical analysis is discussed in Section 3 to reflect the impact of the key factors on the stable strategy. Finally, based on a numerical simulation, the corresponding policy suggestions are presented in Section 4.

## 2. Evolutionary Model of the Government and Construction Enterprise

### 2.1. Model Assumption and Establishment

As the construction industry is the main source of global carbon emissions, the government should pay sufficient attention to policy making. A low-carbon policy aims to reduce the carbon emissions and energy consumption. Prefabrication is an important low-carbon production method in the construction industry. However, in the construction process of a construction project, it is challenging for the government to directly identify whether the construction enterprise (hereinafter indicated by ‘project contractor’) has adopted prefabrication. The project contractors, in turn, are risk averse and seek to maximise their own benefit. As a result, low-carbon behaviour faces uncertainties. Therefore, effective policies should be made to regulate the project contractors’ behaviour.

This study aims to analyse the strategic evolution of the project contractors’ behaviour in adopting prefabrication driven by the government’s low-carbon policy. The government and project contractors are considered the two main players in the model. In the construction industry, the government usually encourages project contractors to adopt prefabrication through initiatives such as subsidies, tax policies, and regulations. However, project contractors tend to adopt conventional construction owing to high initial investment and complex technological innovation of prefabrication. As the government and project contractors are usually bounded by rationality, it is challenging for them, who are constrained by their cognitive level and reasoning ability, to make optimal decisions for the first time. The government and project contractors will adjust the strategies dynamically based on the limited information and others’ behaviour. Therefore, an evolutionary model is constructed to explore the dynamic behaviour of the government and a contractor and the effectiveness of their strategies.

In the model, the following assumptions are made. The government has two strategy options. The first option is to implement low-carbon regulatory policies (hereinafter indicated by ‘Regulate’), including subsidies for prefabrication and carbon tax for all production methods. The second option is to practice a ‘laissez faire’ policy to expect the project contractor to choose the production method based on the market conditions (hereinafter indicated by ‘No-regulate’). Similarly, the project contractor also faces two strategy options, i.e., ‘Implement’ or ‘No-implement’, implying that the project contractor chooses prefabricated or conventional construction, respectively. Assuming that x represents the possibility of the government to take the regulate strategy, then the possibility of taking the no-regulate strategy is 1−x. Similarly, y represents the possibility of the project contractor to take the implement strategy; then, the possibility of taking the no-implement strategy is 1−y.

It is further assumed that the government adopts a low-carbon regulatory policy based on the carbon emissions of the constructed project. Let $e_{L}$ be the carbon emissions of the project constructed using prefabricated production methods. The carbon emissions of conventional construction are $e_{H}$. Let $e_{0}\;$ be the carbon emissions of low-carbon construction projects certified by the government, such as the definition and evaluation standards of low-carbon construction projects proposed in China National Standard ‘Green Building Evaluation Standards’ (GB/T50378-2019), the United States LEED green building evaluation system, and the British BREEAM green building evaluation system. We then have $e_{L}\leq e_{0}\leq e_{H}$. Moreover, under the low-carbon regulatory policy implemented by the government, to promote project contractors to adopt prefabricated production behaviours, the government implements a carbon tax-free policy and subsidises project contractors for prefabricated production behaviours. Assuming that the subsidy coefficient is γ, the number of subsidies obtained by the project contractor for the prefabricated production behaviour is $\gamma \left(e_{0}-e_{L}\right)$. The government levies a carbon tax on project contractors with a tax rate of t. Suppose that the cost of the government-implemented low-carbon regulatory policies is $C_{g}$.

The following assumptions mainly include the costs and utilities considered in the model. The direct economic benefit obtained by the project contractor using the prefabricated production method is $\pi_{1}$, and the direct economic benefit of the conventional construction is $\pi_{2}$. However, additional initial investment for implementing prefabricated construction is needed, such as purchasing new specialised equipment and moulds, technological innovation, and design changes [29], denoted by $C_{k}$. The government can gain environmental values $u_{1}\;$ when the project contractor adopts prefabricated construction and $u_{2}$ when the project contractor adopts conventional construction. Project construction will have negative external effects on the environment. As a result, the government’s environmental governance investment is $w_{L}$ when the project contractor adopts prefabricated construction, and $w_{H}$ when the project contractor adopts conventional construction.

The model parameters, variables and their respective meanings are shown in Table 1.

Based on the descriptions above, the payoff matrix between the governments and the contractors can be obtained, as shown in Table 2.

### 2.2. Model Solution and Analysis

Let $E_{g1}$ and $E_{g2}$ represent the expected payoffs of ‘adopt hybrid policy’ and ‘not adopt hybrid policy’ for the government, respectively. Consequently, $E_{g1}$ and $E_{g2}$ are as follows:

(1) $$E_{g1}=y\left[u_{1}-\gamma \left(e_{0}-e_{L}\right)+te_{L}-w_{L}-C_{g}\right]+\left(1-y\right)\left(u_{2}+te_{H}-w_{H}-C_{g}\right)$$

(2) $$E_{g2}=y\left(u_{1}-w_{L}\right)+\left(1-y\right)\left(u_{2}-w_{H}\right)$$

The average payoff of the government, denoted as $\bar{E_{g}}$, is as follows:

(3) $$\bar{E_{g}}=xE_{g1}+\left(1-x\right)E_{g2}$$

Similarly, let $E_{c1}^{1}$ and $E_{c2}^{1}$ be the expected payoffs of the contractor who takes different strategies (prefabricated or conventional construction, respectively). Consequently, $E_{c1}^{1}$ and $E_{c2}^{1}$ are as follows:

(4) $$E_{c1}=x\left[\pi_{1}+\gamma \left(e_{0}-e_{L}\right)-te_{L}-C_{k}\right]+\left(1-x\right)\left(\pi_{1}-C_{k}\right)$$

(5) $$E_{c2}=x\left(\pi_{2}-te_{H}\right)+\left(1-x\right)\left(\pi_{2}\right)$$

The average payoff of the contractor, denoted as $\bar{E_{c}}$, is as follows:

(6) $$\bar{E_{c}}=yE_{c1}+\left(1-y\right)E_{c2}$$

According to the Malthusian dynamic equation, the replicator dynamic system is treated as a combination of the replicator dynamic equations of the government and contractor.

(7) $$\{\begin{array}{c} F\left(x\right)=\frac{dx}{dt}=x\left(1-x\right)\left[te_{H}-C_{g}-y\left[\gamma \left(e_{0}-e_{L}\right)+t\left(e_{H}-e_{L}\right)\right]\right]\\ F\left(y\right)=\frac{dy}{dt}=y\left(1-y\right)\left[x\left[\gamma \left(e_{0}-e_{L}\right)+t\left(e_{H}-e_{L}\right)\right]-\pi_{2}-C_{k}+\pi_{1}\right] \end{array}$$

According to the stability theorem of differential equations, let F(x)=0 and F(θ); then, five local equilibrium points can be obtained: O(0, 0), A(0, 1), B(1, 0), C(1, 1), D($x^{*}$, $y^{*}$), with $x^{*}=\frac{\pi_{2}-\pi_{1}+C_{k}}{\gamma \left(e_{0}-e_{L}\right)+t\left(e_{H}-e_{L}\right)}$, $y^{*}=\frac{te_{H}-C_{g}}{\gamma \left(e_{0}-e_{L}\right)+t\left(e_{H}-e_{L}\right)}$.

In order to analyse the stability of the equilibrium points, the Jacobian matrix of the above-defined replicator dynamic system is established as follows:

(8) $$J=\left[\begin{array}{cc} a_{11} & a_{12}\\ a_{21} & a_{22} \end{array}\right]$$

Correspondingly,

(9) $$a_{11}=\left(1-2x\right)\left[te_{H}-C_{g}-y\left[\gamma \left(e_{0}-e_{L}\right)+t\left(e_{H}-e_{L}\right)\right]\right]$$

(10) $$a_{12}=-x\left(1-x\right)\left[\gamma \left(e_{0}-e_{L}\right)+t\left(e_{H}-e_{L}\right)\right]$$

(11) $$a_{21}=y\left(1-y\right)\left[\gamma \left(e_{0}-e_{L}\right)+t\left(e_{H}-e_{L}\right)\right]$$

(12) $$a_{22}=\left(1-2y\right)\left[x\left[\gamma \left(e_{0}-e_{L}\right)+t\left(e_{H}-e_{L}\right)\right]-\pi_{2}-C_{k}+\pi_{1}\right]$$

The determinant (det) and trace (tr) of J are as follows:

(13) $$\begin{array}{cc} \det J=\left(1-2x\right) & \left(1-2y\right)\left[te_{H}-C_{g}-y\left[\gamma \left(e_{0}-e_{L}\right)+t\left(e_{H}-e_{L}\right)\right]\right]\left[x\left[\gamma \left(e_{0}-e_{L}\right)+t\left(e_{H}-e_{L}\right)\right]-\pi_{2}-C_{k}+\pi_{1}\right]\\ & \;\;+x\theta \left(1-x\right)\left(1-y\right){\left[\gamma \left(e_{0}-e_{L}\right)+t\left(e_{H}-e_{L}\right)\right]}^{2} \end{array}$$

(14) $$\begin{array}{cc} \mathrm{tr}J=(1-2x) & [te_{H}-C_{g}-y\left[\gamma \left(e_{0}-e_{L}\right)+t\left(e_{H}-e_{L}\right)\right]]+(1\\ \; & -2y)\left[x\left[\gamma \left(e_{0}-e_{L}\right)+t\left(e_{H}-e_{L}\right)\right]-\pi_{2}-C_{k}+\pi_{1}\right] \end{array}$$

The stability conditions of the replicator dynamic equations are divided into nine scenarios. The stability of each equilibrium point in each scenario is summarised in Table 3. Here, ‘+’ denotes that the values of detJ or tr J exceed 0, ‘−’ denotes that the values of detJ or tr J are less than 0, and ‘N’ denotes that the value of tr J has uncertainty. If detJ>0 and tr J<0, the evolutionary stable strategy (ESS) exists. Under ESS, neither the government nor the project contractor can achieve greater benefits by changing own strategies, thus forming a stable behavioural state.

Scenario 1: When $\pi_{1}-C_{k}>\pi_{2}$ and $te_{H}<C_{g}$, the difference between the direct economic benefit obtained by adopting prefabrication and the initial investment is more than the direct economic benefit obtained by implementing conventional construction. In addition, the carbon tax imposed by the government on the contractor who adopts conventional construction is less than the regulatory cost; (0, 1) is an ESS of the replicator dynamic system. The equilibrium behaviour strategy is ‘Do not regulate’ and ‘Implement’.

Scenario 2: When $\pi_{1}-C_{k}>\pi_{2}$ and $0<te_{H}-C_{g}<\gamma \left(e_{0}-e_{L}\right)+t\left(e_{H}-e_{L}\right)$, the difference between the direct economic benefit obtained by adopting prefabrication and the initial investment is more than the direct economic benefit obtained by implementing conventional construction. The carbon tax imposed by the government on the contractor who adopts conventional construction is less than the regulatory cost. In addition, the carbon taxes the government collects from prefabrication after deducting the regulatory fees and prefabrication subsidies are less than 0. Here, (0, 1) is an ESS of the replicator dynamic system. The equilibrium behaviour strategy is ‘Do not regulate’ and ‘Implement’.

Scenario 3: When $\pi_{1}-C_{k}>\pi_{2}$ and $te_{H}-C_{g}>\gamma \left(e_{0}-e_{L}\right)+t\left(e_{H}-e_{L}\right)$, that is the difference between the direct economic benefit obtained by adopting prefabrication and the initial investment is more than the direct economic benefit obtained by implementing conventional construction. In addition, the carbon taxes the government collects from prefabrication after deducting the regulatory fees and prefabrication subsidies are more than 0. Here, (1, 1) is an ESS of the replicator dynamic system. The equilibrium behaviour strategy is ‘Regulate’ and ‘Implement’.

Scenario 4: When $0<\pi_{2}+C_{k}-\pi_{1}<\gamma \left(e_{0}-e_{L}\right)+t\left(e_{H}-e_{L}\right)$ and $te_{H}<C_{g}$, the difference between the direct economic benefit obtained by adopting prefabrication and the initial investment is less than the direct economic benefit obtained by implementing conventional construction. The profit of the project contractor by implementing prefabrication exceeds that by implementing conventional construction. In addition, the carbon tax imposed by the government on the contractor who adopts conventional construction is less than the regulatory cost; (0, 0) is an ESS of the replicator dynamic system. The equilibrium behaviour strategy is ‘Do not regulate’ and ‘Do not implement’.

Scenario 5: When $0<\pi_{2}+C_{k}-\pi_{1}<\gamma \left(e_{0}-e_{L}\right)+t\left(e_{H}-e_{L}\right)$ and $0<te_{H}-C_{g}<\gamma \left(e_{0}-e_{L}\right)+t\left(e_{H}-e_{L}\right)$, the difference between the direct economic benefit obtained by adopting prefabrication and the initial investment is less than the direct economic benefit obtained by implementing conventional construction. The profit of the project contractor by implementing prefabrication exceeds that by implementing conventional construction. In addition, the carbon tax imposed by the government on the contractor who adopts conventional construction is less than the regulatory cost. The carbon taxes the government collects from prefabrication after deducting the regulatory fees and prefabrication subsidies are less than 0; the system has no ESS.

Scenario 6: When $0<\pi_{2}+C_{k}-\pi_{1}<\gamma \left(e_{0}-e_{L}\right)+t\left(e_{H}-e_{L}\right)$ and $te_{H}-C_{g}>\gamma \left(e_{0}-e_{L}\right)+t\left(e_{H}-e_{L}\right)$, the difference between the direct economic benefit obtained by adopting prefabrication and the initial investment is less than the direct economic benefit obtained by implementing conventional construction. The profit of the project contractor by implementing prefabrication exceeds that by implementing conventional construction. In addition, the carbon taxes the government collects from prefabrication after deducting the regulatory fees and prefabrication subsidies are more than 0. Here, (1, 1) is an ESS of the replicator dynamic system. The equilibrium behaviour strategy is ‘Regulate’ and ‘Implement’.

Scenario 7: When $\pi_{2}+C_{k}-\pi_{1}>\gamma \left(e_{0}-e_{L}\right)+t\left(e_{H}-e_{L}\right)$ and $te_{H}<C_{g}$, the profit of the project contractor by implementing prefabrication is less than that by implementing conventional construction. In addition, the carbon tax imposed by the government on the contractor who adopts conventional construction is less than the regulatory cost. Here, (0, 0) is an ESS of the replicator dynamic system. The equilibrium behaviour strategy is ‘Do not regulate’ and ‘Do not implement’.

Scenario 8: When $\pi_{2}+C_{k}-\pi_{1}>\gamma \left(e_{0}-e_{L}\right)+t\left(e_{H}-e_{L}\right)$ and $0<te_{H}-C_{g}<\gamma \left(e_{0}-e_{L}\right)+t\left(e_{H}-e_{L}\right)$, the profit of the project contractor by implementing prefabrication is less than that by implementing conventional construction. The carbon tax imposed by the government on the contractor who adopts conventional construction is less than the regulatory cost. In addition, the carbon taxes the government collects from prefabrication after deducting the regulatory fees and prefabrication subsidies are less than 0. Here, (1, 0) is an ESS of the replicator dynamic system. The equilibrium behaviour strategy is ‘Regulate’ and ‘Do not implement’.

Scenario 9: When $\pi_{2}+C_{k}-\pi_{1}>\gamma \left(e_{0}-e_{L}\right)+t\left(e_{H}-e_{L}\right)$ and $te_{H}-C_{g}>\gamma \left(e_{0}-e_{L}\right)+t\left(e_{H}-e_{L}\right)$, the profit of the project contractor by implementing prefabrication is less than that by implementing conventional construction. In addition, the carbon taxes the government collects from prefabrication after deducting the regulatory fees and prefabrication subsidies exceed 0. Here, (1, 0) is an ESS of the replicator dynamic system. The equilibrium behaviour strategy is ‘Regulate’ and ‘Do not implement’.

From Table 3 and the above-discussed scenario analysis, the following conclusions can be obtained.

Conclusion 1. When the government’s regulatory cost to implement the low-carbon policy exceeds the tax paid by contractors using conventional construction, that is as long as $te_{H}<C_{g}$, the evolution direction of the government is to take the do not regulate strategy. This is because of the need to achieve a profit balance for the government. Otherwise, the government is reluctant to implement low-carbon policies to encourage prefabricated production.

Conclusion 2. When the regulatory cost of implementing a low-carbon policy by the government is less than the difference between the tax paid by the contractor for prefabrication and the government subsidy, that is as long as $C_{g}<\gamma \left(e_{0}-e_{L}\right)-te_{L}$, the evolution direction of the government is to take the regulate strategy.

Conclusion 3. When the difference between the direct economic benefit obtained by adopting prefabrication and the initial investment exceeds the direct economic benefit obtained by implementing conventional construction, that is as long as $\pi_{1}-C_{k}>\pi_{2}$, the evolution direction of the project contractor is to take the implement prefabrication strategy. Moreover, when $0<\pi_{2}+C_{k}-\pi_{1}<\gamma \left(e_{0}-e_{L}\right)+t\left(e_{H}-e_{L}\right)$ and $te_{H}-C_{g}>\gamma \left(e_{0}-e_{L}\right)+t\left(e_{H}-e_{L}\right)$, the system evolves in a positive direction and achieves a stable state at (1,1). Thus, as long as the government’s low-carbon policy makes up for the profit gap between prefabricated and conventional construction for project contractors so that the profit of prefabrication exceeds that of conventional construction, the contractors are willing to implement prefabrication strategies.

Conclusion 4. When the net profit of the project contractor by implementing prefabrication is less than that by implementing conventional construction under a low-carbon policy, that is as long as $\pi_{2}+C_{k}-\pi_{1}>\gamma \left(e_{0}-e_{L}\right)+t\left(e_{H}-e_{L}\right)$, the evolution direction of the project contractor is to take the implement conventional construction strategy.

## 3. Numerical Analysis

To reflect the impact of the key factors on the stable strategy further, a numerical analysis was conducted herein. Nanjing was selected as the first batch of prefabricated building demonstration cities in 2017. Under the promotion of national policies, the Nanjing Municipal Government issued the ‘Notice on the Implementation Opinions on Further Promoting the Development of Prefabricated Buildings’, proposing that, by 2020, the city’s prefabricated buildings will account for more than 30% of the proportion of new buildings. According to statistics, the proportion of newly started prefabricated buildings in the new construction area of the year has increased from 10.34% in 2016 to 38.72% in 2020. The carbon peak and carbon neutral policies were proposed in 2020. Nanjing has launched a number of policies to promote prefabricated buildings in response to carbon policies. Therefore, a prefabricated residential project in Nanjing city in China was selected as the reference for the initial values of the parameters and variables. Based on a survey and an interview, the actual data were simplified without affecting the results. The initial value used in the case analysis was set to $\pi_{1}=3000$, $\pi_{2}=2830$, $C_{k}=200$, $C_{g}=1010$, $e_{L}=23.4$, $e_{H}=25.8$, $e_{0}=25$, γ=14, and t=40.

### 3.1. Influence of the Regulatory Cost on the Evolutionary System

**Figure 1:**
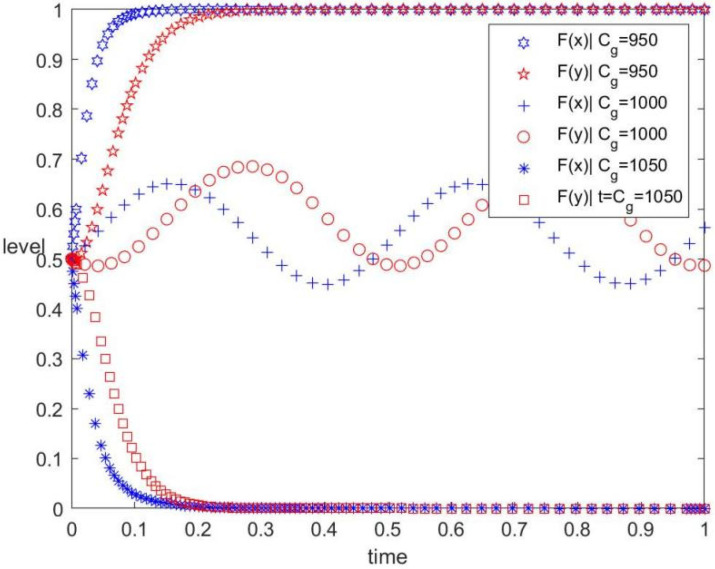
Evolution track under different regulatory costs.

The $C_{g}$ values are 950, 1000, and 1050, corresponding to low, medium, and high regulatory costs for the government, respectively. The values of other parameters are unchanged. The evolution track of both the government and the contractors in the replication dynamic system under different regulatory costs is shown in Figure 1. The figure also displays the influence of the regulatory cost on the evolutionary game. As can be observed, when $C_{g}=950$, the evolutionary stable point of the system is (1, 1). When $C_{g}=1000$, the system has no evolutionary stable point. When $C_{g}=1050$, the evolutionary stable point of the system is (0, 0). Figure 1 shows that the government will transfer the strategy from ‘regulate’ to ‘uncertainty’, then to ‘no-regulate’ after increasing the regulatory cost, whereas the evolution trend of contractor’s behaviour strategy is the same.

As shown in Figure 1, the regulatory cost is an important factor influencing the behaviour strategy selection of both the government and the contractors. In order to regulate low-carbon policies and to improve disclosure and sharing of information on the green behaviour of construction enterprises, the government should increase the investment in policy formulation, promotion, and regulation and the establishment of the information platform. However, excessive investment will increase the financial burden on the government and reduce confidence. When the government increases the regulatory cost blindly, the contractor will also be cautious about the behaviour strategy.

### 3.2. Influence of the Initial Investment on the Evolutionary System

**Figure 2:**
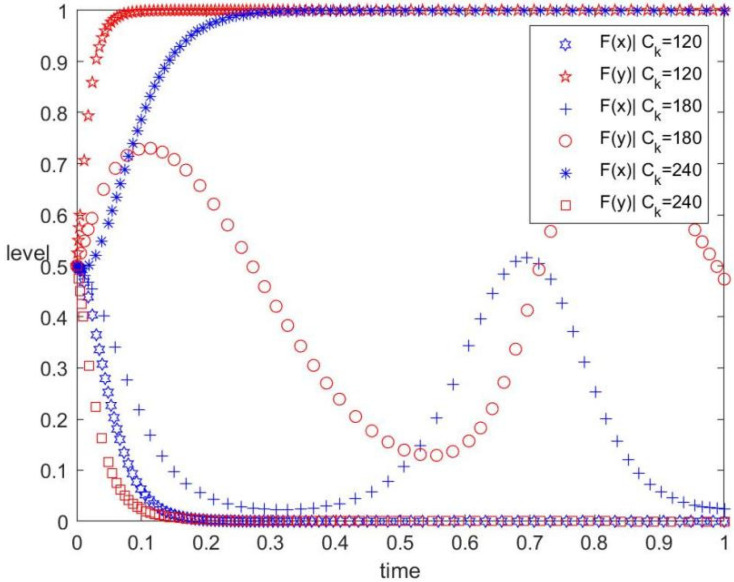
Evolution track under different additional initial investment.

The $C_{k}$ values are 120, 180, and 240, corresponding to low, medium, and high investment intensities by the contractors, respectively. The values of other parameters are unchanged. The evolution track of both the government and the contractors in the replication dynamic system under different investment intensities is shown in Figure 2. The figure also displays the influence of additional initial investment of the contractors into prefabrication on the evolutionary game. As can be observed, when the initial investment is low, the contractors evolve to prefabricated construction. This is an ideal state where the contractors are willing to implement prefabricated behaviour no matter what the government’s behaviour is. However, with an increasing initial investment, the behaviour of the contractors will be chaotic even under the government’s policy subsidy. When the government’s subsidies are not sufficient to make up for this large initial investment, the contractors will continue to implement conventional construction methods.

As shown in Figure 2, the additional initial investment is an important factor influencing the behaviour strategy selection of contractors. The additional initial investment mainly attributes to the high requirement on the degree of standardisation, specialisation, and scale of prefabricated components, such as equipment purchase, reform of the production line, and technological innovation. Therefore, the promotion of a mature prefabrication market and low-carbon prefabricated technologies should be considered.

### 3.3. Influence of the Subsidy Intensity on the Evolutionary System

**Figure 3:**
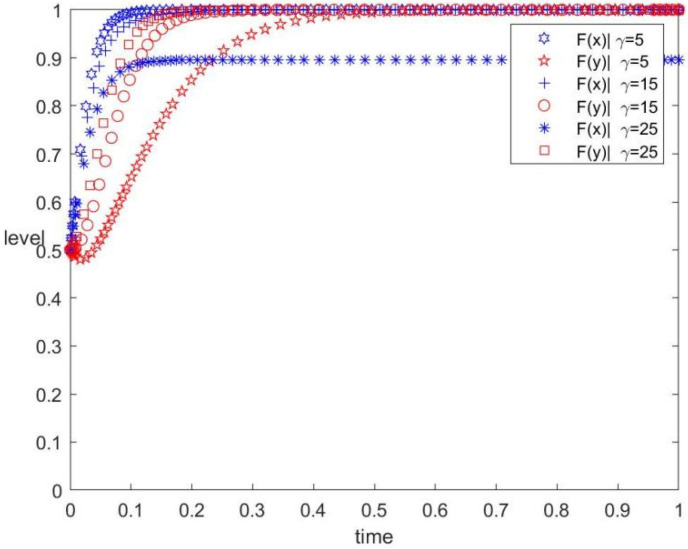
Evolution track under different subsidy intensities.

The γ values are 5, 15, and 25, corresponding to low, medium, and high subsidy intensities, respectively. The values of other parameters are unchanged. The evolution track of both the government and the contractors in the replication dynamic system under different subsidy intensities is shown in Figure 3. The figure also displays the influence of the subsidy intensity of the government on the evolutionary game. As can be observed, in the process of implementing low-carbon subsidy policies by the government to guide market development, the contractors evolve to implement prefabricated construction. The system will remain stable at (1, 1). Moreover, the higher the subsidy intensity, the faster the contractor tends to implement the prefabricated behaviour.

As shown in Figure 3, the subsidy intensity from the government has a positive impact on the strategy selection of the contractors. When the government provides a low subsidy intensity, the contractors evolve to implement conventional construction because of the high initial investment. When the government increases the subsidy intensity moderately, the contractors are willing to implement prefabricated construction because they can receive sufficient financial support from the government to offset the additional investment in prefabrication. In contrast, when high subsidy intensity puts huge financial pressure on the government, the effectiveness of the subsidy policy will be reduced. Therefore, blindly increasing the intensity of subsidies is not appropriate for the government because the contractors will choose their behaviour strategies more carefully, owing to the government’s financial pressure.

### 3.4. Influence of the Tax Intensity on the Evolutionary System

**Figure 4:**
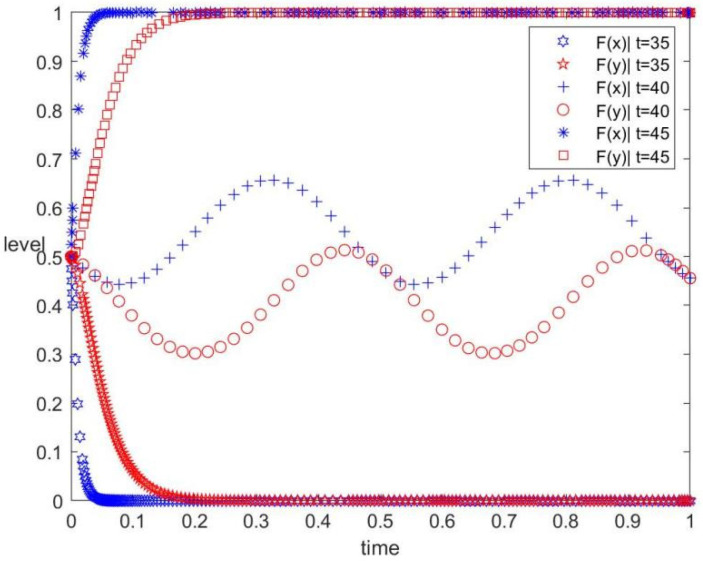
Evolution track under different tax intensities.

The t values are 35, 40, and 45, corresponding to low, medium, and high tax intensities, respectively. The values of other parameters are unchanged. The evolution track of both the government and the contractors in the replication dynamic system under different tax intensities is shown in Figure 4. The figure also displays the influence of the tax intensity of the government on the evolutionary game. As can be observed, when t=35, the evolutionary stable point of the system is (0, 0). When t=40, the system has no evolutionary stable point. When t=45, the evolutionary stable point of the system is (1, 1). Figure 4 shows that the contractors will transfer their strategy from ‘do not implement’ to ‘uncertainty’, then to ‘implement’ after increasing the tax intensity, whereas the evolution trend of the government’s behaviour strategy is the same.

As shown in Figure 4, when the government provides a low tax intensity, the contractors evolve to implement conventional construction to obtain higher profits. When the government increases the tax intensity, the contractors will tend to implement prefabricated construction to avoid high taxes. When the carbon tax completely offsets the additional income brought by conventional construction, the contractors will implement prefabricated construction stably. Therefore, an effective taxation mechanism and punishment mechanism can effectively increase the enthusiasm of construction enterprises for green behaviour.

## 4. Conclusions

In the low-carbon era, the problem of promoting prefabricated green construction has become highly relevant to both practitioners in the construction industry and the government. In order to analyse the green construction behaviour tendency under a low-carbon policy, an evolutionary game model between the government and construction enterprise was developed in this study. The payoff matrix under different strategies was proposed, and the replicator dynamic system was formulated. Subsequently, nine scenarios were summarised, and four conclusions were proposed. The influence of the key factors on the behaviour strategies of both the government and the construction enterprise was further investigated through numerical analysis.

This study makes two contributions to the existing literature. First, this study analyses the comprehensive impact of incentives and carbon tax on prefabrication implementation, Moreover, it proposes a method for verifying the effectiveness of diversified policies in guiding the behaviour of construction enterprises. Second, this study reveals the dynamic game mechanism between the government and construction enterprises under several key factors, which can help the government adjust strategy dynamically to control the evolution direction of the system. Based on the evolutionary game analysis, the following management insights were concluded.

(1) The government’s low-carbon policy has an effective guiding effect on the contractor’s prefabrication behaviour. Therefore, the government must continue to play a leading role in the contractors’ adoption of low-carbon prefabricated construction. Moreover, the government should establish an institutional framework that integrates subsidy and tax mechanisms [30], such as subsidies for reduced carbon emissions and taxes on carbon emissions. The government should also consider the market maturity, local economic level, technical level, resources, and culture. Furthermore, the government needs to adjust the policies and guidance in response to the dynamic behaviour of the construction enterprise. The government’s policies not only satisfactorily compensate for the market failures but also avoid fiscal overruns.

(2) The subsidy and tax intensity are important factors affecting the implementation of prefabrication by the construction enterprise. The higher the subsidy and the carbon tax, the stronger the local government’s motivation to guide the development of the prefabricated construction market. Appropriate subsidy and tax intensity should be adopted by the government to achieve a regulated and stable market. From Figure 3, although higher subsidy incentives indirectly release relevant positive signals to the market, the government’s increased financial subsidies will not necessarily increase the enthusiasm of construction enterprises. On the contrary, construction enterprises will feel a sense of crisis due to high financial pressure. In addition, from Figure 4, high tax intensity can also hinder construction enterprises from entering the market. Therefore, the government should avoid blindly increasing financial subsidies and the tax rate [20].

(3) Additional initial investment directly affects the willingness of the construction enterprise to implement prefabrication (Figure 2). Therefore, the government can reduce this cost through a series of measures. For example, the government cannot only strengthen the incentives in compensating prefabricated construction but also regulate the market price of prefabricated components through the whole supply chain management. In addition, the government can also encourage construction enterprises to expand production. The reason is that when the scale reaches a certain level, the equipment depreciation and the amortised cost can reduce the initial investment of prefabrication.

(4) The regulatory cost directly affects the willingness of the government to implement low-carbon policies (Figure 1). The probability of applying a hybrid policy of subsidies and taxation will decrease as the regulatory cost for the government increases, and vice versa. Therefore, the government should not only improve the government’s ability to supervise carbon emissions but also strive for a gradual reduction of regulatory costs and improvement in the regulatory efficiency and effectiveness by formulating a complete information platform [31].

(5) In different periods of market development, the government should adopt different policies. The subsidy policy should be biased towards the prefabricated market that needs to improve its maturity. Therefore, in the early stage of market development, the government should focus on providing sufficient subsidies to cover the high initial cost of prefabricated enterprises and to guide the behaviour of enterprises through the carbon tax. In the mid-term of market development, attention should be paid to dynamic adjustment of policies to ensure their effectiveness. After the market has matured, similar to the snowballing phenomenon, the construction enterprise will continue to implement prefabrication regardless of the government’s policies. At this stage, the market mechanism can play a leading role.

This study has the following limitations. It only analyses the evolutionary behaviour under mixed policies of tax and subsidy. However, diversity policy should be considered in the future. The impact of the dynamics and higher incentive and penalty measures on the system, such as reputational incentives, should also be further investigated.

## Figures and Tables

**Table 1:** Parameters and variables.

**Parameters**	**Descriptions**
$e_{L}$	Carbon emissions when the project contractor implements prefabrication
$e_{H}$	Carbon emissions when the project contractor implements conventional construction
$e_{0}$	Carbon emissions of low-carbon construction projects certified by the government
γ	The subsidy coefficient for the prefabricated construction
t	Carbon tax rate on the project contractors
$\pi_{1}$	Direct economic benefit obtained by the project contractor adopting prefabricated construction
$\pi_{2}$	Direct economic benefit obtained by the project contractor adopting conventional construction
$C_{g}$	The cost of the government-implemented low-carbon regulatory policies
$C_{k}$	Additional initial investment for implementing prefabricated construction
$w_{L}$	Government’s environmental governance investment when the project contractor adopts prefabricated construction
$w_{H}$	Government’s environmental governance investment when the project contractor adopts conventional construction
$u_{1}$	Government’s environmental values when the project contractor adopts prefabrication
$u_{2}$	Government’s environmental values when the project contractor adopts conventional construction
**Variables**	**Descriptions**
x	Probability that the government adopts low-carbon regulatory policies
y	Probability that the contractor adopt prefabrication

**Table 2:** Payoff matrix between the governments and the contractors.

Government	Contractors	
	Implement	No-Implement
Regulate	$u_{1}-\gamma \left(e_{0}-e_{L}\right)+te_{L}-w_{L}-C_{g}$, $\pi_{1}+\gamma \left(e_{0}-e_{L}\right)-te_{L}-C_{k}$	$u_{2}+te_{H}-w_{H}-C_{g}$, $\pi_{2}-te_{H}$
Do not regulate	$u_{1}-w_{L}$, $\pi_{1}-C_{k}$	$u_{2}-w_{H}$, $\pi_{2}$

**Table 3:** Stability analysis of nine scenarios.

**Equilibrium Point**	$(a)\;\pi_{1}>\pi_{2}+C_{k}$								
	$te_{H}<C_{g}$			$0<te_{H}-C_{g}<$ $\gamma \left(e_{0}-e_{L}\right)+t\left(e_{H}-e_{L}\right)$			$te_{H}-C_{g}>$ $\gamma \left(e_{0}-e_{L}\right)+t\left(e_{H}-e_{L}\right)$		
	detJ	tr J	**State**	detJ	tr J	**State**	detJ	tr J	**State**
(0, 0)	−	N	Saddle point	+	+	Instability point	+	+	Instability point
(0, 1)	+	−	ESS	+	−	ESS	−	N	Saddle point
(1, 0)	+	+	Instability point	−	N	Saddle point	−	N	Saddle point
(1, 1)	−	N	Saddle point	−	N	Saddle point	+	−	ESS
$(x^{*}$$,\;y^{*}$)	Meaningless			Meaningless			Meaningless		
**Equilibrium Point**	$(b)\;0<\pi_{2}+C_{k}-\pi_{1}<\gamma \left(e_{0}-e_{L}\right)+t\left(e_{H}-e_{L}\right)$								
	$te_{H}<C_{g}$			$0<te_{H}-C_{g}<$ $\gamma \left(e_{0}-e_{L}\right)+t\left(e_{H}-e_{L}\right)$			$te_{H}-C_{g}>$ $\gamma \left(e_{0}-e_{L}\right)+t\left(e_{H}-e_{L}\right)$		
	detJ	tr J	**State**	detJ	tr J	**State**	detJ	tr J	**State**
(0, 0)	+	−	ESS	−	N	Saddle point	−	N	Saddle point
(0, 1)	−	N	Saddle point	−	N	Saddle point	+	+	Instability point
(1, 0)	+	+	Instability point	−	N	Saddle point	−	N	Saddle point
(1, 1)	−	N	Saddle point	−	N	Saddle point	+	−	ESS
$(x^{*}$$,\;y^{*}$)	Meaningless			+	0	Central point	Meaningless		
**Equilibrium Point**	$(c)\;\pi_{2}+C_{k}-\pi_{1}>\gamma \left(e_{0}-e_{L}\right)+t\left(e_{H}-e_{L}\right)$								
	$te_{H}<C_{g}$			$0<te_{H}-C_{g}<$ $\gamma \left(e_{0}-e_{L}\right)+t\left(e_{H}-e_{L}\right)$			$te_{H}-C_{g}>$ $\gamma \left(e_{0}-e_{L}\right)+t\left(e_{H}-e_{L}\right)$		
	detJ	tr J	**State**	detJ	tr J	**State**	detJ	tr J	**State**
(0, 0)	+	−	ESS	−	N	Saddle point	−	N	Saddle point
(0, 1)	−	N	Saddle point	−	N	Saddle point	+	+	Instability point
(1, 0)	−	N	Saddle point	+	−	ESS	+	−	ESS
(1, 1)	−	N	Saddle point	+	+	Instability point	−	N	Saddle point
$(x^{*}$$,\;y^{*}$)	Meaningless			Meaningless			Meaningless		
